# Supplementary material for: Endothelial cells regulate alveolar morphogenesis by constructing basement membranes acting as a scaffold for myofibroblasts
Source: Nat Commun. 2024 Mar 4;15:1622. doi: 10.1038/s41467-024-45910-y (PMC10912381; doi:10.1038/s41467-024-45910-y)
Supplement: Supplementary file 1 — Supplementary Information [file 41467_2024_45910_MOESM1_ESM.pdf]

SUPPLEMENTARY INFORMATION

Endothelial cells regulate alveolar morphogenesis by constructing basement membranes acting as a scaffold for myofibroblasts

Haruko Watanabe-Takano, Katsuhiro Kato, Eri Oguri-Nakamura, Tomohiro Ishi, Koji Kobayashi, Takahisa Murata, Koichiro Tsujikawa, Takaki Miyata, Yoshiaki Kubota, Yasuyuki Hanada, Koichi Nishiyama, Tetsuro Watabe, Reinhard Fässler, Hirotaka Ishii, Naoki Mochizuki, Shigetomo Fukuhara

INVENTORY OF SUPPLEMENTARY INFORMATION

Supplementary Figures 1-7

Supplementary Tables 1-3

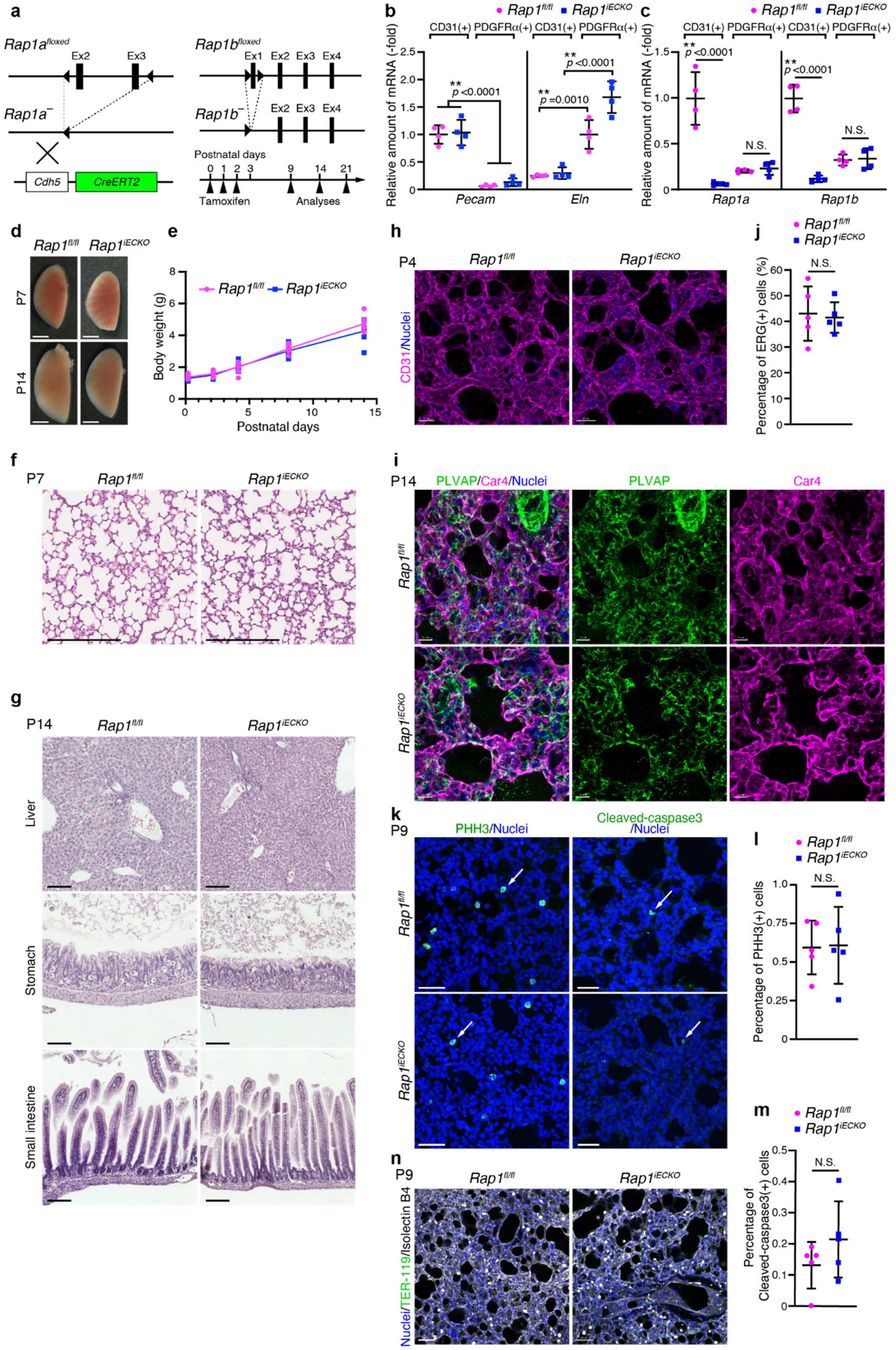
a
Rap1afloxed
Ex2
Ex3
Rap1a−
Cdh5
CreERT2
Rap1bfloxed
Ex1
Ex2
Ex3
Ex4
Rap1b−
Postnatal days
0 1 2 3 9 14 21
Tamoxifen
Analyses
b
Rap1fl/fl
Rap1iECKO
CD31(+) PDGFRα(+) CD31(+) PDGFRα(+)
Relative amount of mRNA (-fold)
p <0.0001
p =0.0010
Pecam
Eln
c
Rap1a
Rap1b
N.S.
d
P7
P14
e
Body weight (g)
Postnatal days
f
P7
g
P14
Liver
Stomach
Small intestine
h
P4
CD31/Nuclei
i
P14
PLVAP/Car4/Nuclei
PLVAP
Car4
j
Percentage of ERG(+) cells (%)
k
P9
PHH3/Nuclei
Cleaved-caspase3 /Nuclei
l
Percentage of PHH3(+) cells
m
Percentage of Cleaved-caspase3(+) cells
n
P9
Nuclei/TER-119/Isolectin B4

Supplementary Fig. 1. Alveolar capillary networks develop normally in *Rap1*^{iECKO} mice.

a, Generation of mice lacking both *Rap1a* and *Rap1b* specifically in ECs (*Rap1*^{iECKO}). Schematic representations of *Rap1a* floxed and knockout alleles (left) and *Rap1b* floxed and knockout alleles (right). In *Rap1a* and *Rap1b* floxed alleles, exons 2 to 3 and exon 1 are flanked by two loxP sites, respectively. To generate *Rap1*^{iECKO} mice, the progenies obtained by crossing the double-floxed *Rap1a/b* mice with the mouse line expressing CreERT2 recombinase under the control of the *Cdh5* promoter (*Cdh5-CreERT2*) were administered with tamoxifen for three consecutive days from P0 through the lactating dam. Analyses were conducted at P9, P14, and P21 as indicated in the Figure legends.

b, Expression levels of *Pecam1* (left) and *Eln* (right) in CD31(+) cells or PDGFRα(+) cells isolated from the lungs of *Rap1*$^{fl/fl}$ and *Rap1*^{iECKO} mice at P9 analyzed by quantitative PCR relative to that of *Actb* (n = 4 mice/each).

c, Expression levels of *Rap1a* (left) and *Rap1b* (right) in CD31(+) cells or PDGFRα(+) cells isolated from the lungs of *Rap1*$^{fl/fl}$ and *Rap1*^{iECKO} mice at P9 are shown as in **b** (n = 4 mice/each).

d, Appearances of the left lungs from *Rap1*$^{fl/fl}$ and *Rap1*^{iECKO} mice at P7 and P14. Note that the *Rap1*^{iECKO} mouse lung is smaller than the control mouse lung at P14, but not at P7.

e, Growth curves of *Rap1*$^{fl/fl}$ and *Rap1*^{iECKO} neonatal mice from 1 to 14 days of age. Data are means ± s.d. (n = 7 mice/each).

f, Images of HE-stained lungs of *Rap1*$^{fl/fl}$ and *Rap1*^{iECKO} mice at P7. Note that alveolar spaces are similar in *Rap1*$^{fl/fl}$ and *Rap1*^{iECKO} mice.

g, Images of HE-stained liver (upper), stomach (middle) and small intestine (lower) of *Rap1*$^{fl/fl}$ and *Rap1*^{iECKO} mice at P14.

h, Confocal z-projection images of distal lungs stained with anti-CD31 antibody (magenta) and DAPI (Nuclei, blue) from *Rap1*$^{fl/fl}$ and *Rap1*^{iECKO} mice at P4. The merged images are shown.

i, Confocal z-projection images for PLVAP (green), Car4 (magenta), and Nuclei (DAPI, blue) in alveoli from *Rap1*$^{fl/fl}$ and *Rap1*^{iECKO} mice at P14.

j, Percentages of ERG(+) cells relative to the total number of cells in *Rap1*$^{fl/fl}$ and *Rap1*^{iECKO} lungs at P9 (n = 5 mice/each).

k, Confocal z-projection images for PHH3 (left, green) and Cleaved-caspase3 (right, green) in alveoli from *Rap1*$^{fl/fl}$ and *Rap1*^{iECKO} mice at P9. Nuclei (blue) were also stained by DAPI.

l, **m**, Percentages of PHH3(+) cells (**l**) and Cleaved-caspase-3(+) cells (**m**) relative to the total number of cells, as in **k** (n = 5 mice/each).

n, Confocal z-projection images for TER119 (green), isolectin B4 (white), and Nuclei

(DAPI, blue) in alveoli from *Rap1*$^{fl/fl}$ and *Rap1*^{iECKO} mice at P9. Note that there is no leakage of TER-119-labeled erythrocytes from alveolar capillaries in the *Rap1*^{iECKO} mouse.

Each dot represents an individual mouse, and data are means ± s.d. (**b**, **c**, **j**, **l**, **m**). N.S., no significance; $^{**}P < 0.01$. Statistical significance was determined by one-way ANOVA followed by Tukey's post-hoc test (**b, c**) and by two tailed Student's *t*-test (**e**, **j, l, m**). Scale bars; 0.5 mm (**d**), 250 µm (**f**), 100 µm (**g**), 20 µm (**i**), 30 µm (**h**, **k**), 50 µm (**n**). Source data are provided as a Source data file.

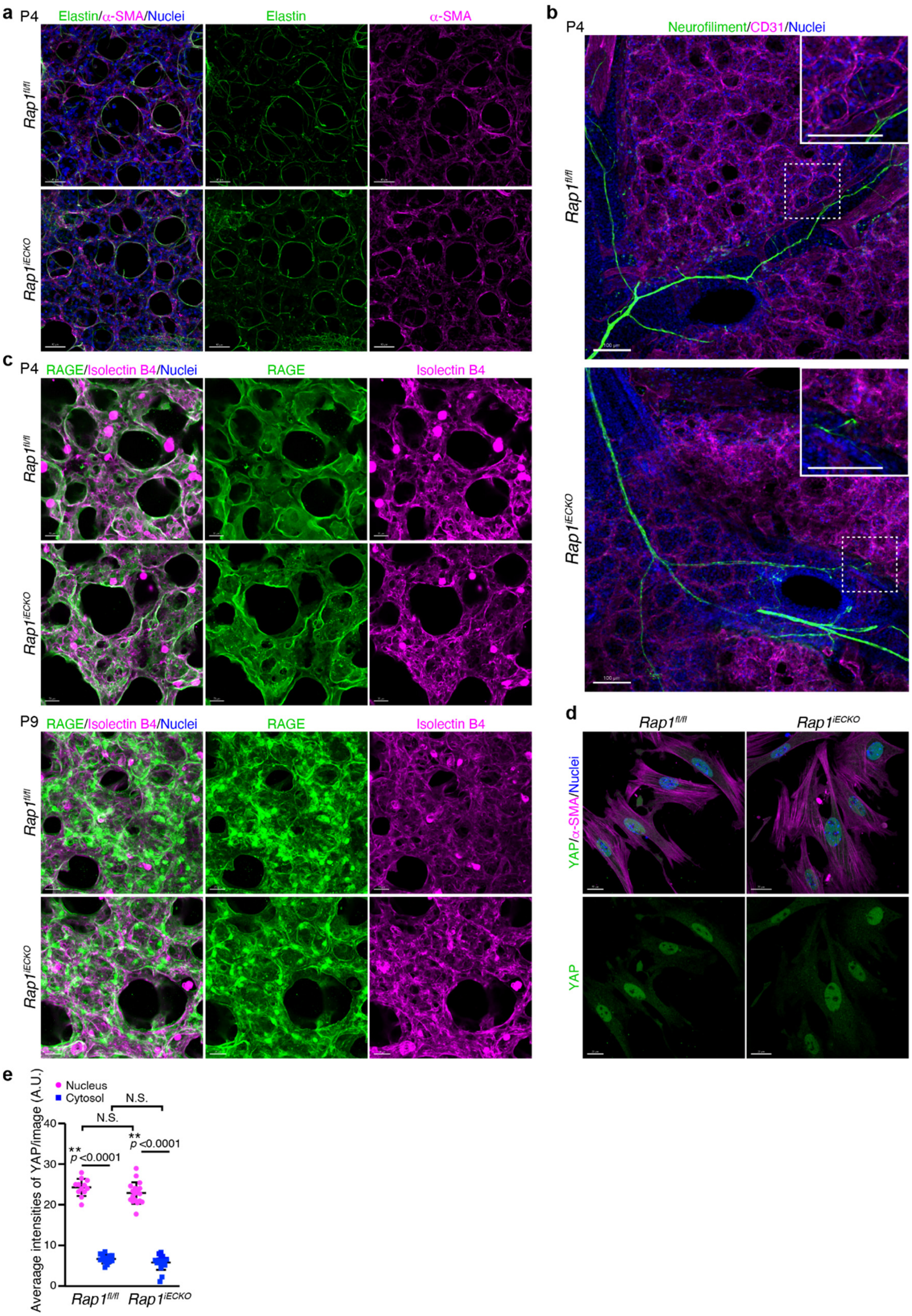
a
P4
Elastin/α-SMA/Nuclei
Elastin
α-SMA
Rap1fl/fl
Rap1iECKO
b
P4
Neurofiliment/CD31/Nuclei
Rap1fl/fl
Rap1iECKO
c
P4
RAGE/Isolectin B4/Nuclei
RAGE
Isolectin B4
P9
RAGE/Isolectin B4/Nuclei
RAGE
Isolectin B4
d
Rap1fl/fl
Rap1iECKO
YAP/α-SMA/Nuclei
YAP
e
Nucleus
Cytosol
N.S.
N.S.
**
p <0.0001
**
p <0.0001
Averaage intensities of YAP/image (A.U.)
40
30
20
10
0
Rap1fl/fl
Rap1iECKO

Supplementary Fig. 2. Alveolar cells are present during postnatal lung development in *Rap1*^{iECKO} mice.

a, Confocal z-projection images for Elastin (green), α-SMA (magenta), and Nuclei (DAPI, blue) in alveoli from *Rap1*$^{fl/fl}$ and *Rap1*^{iECKO} mice at P4.

b, Confocal z-projection images for neurofilament (green), CD31 (magenta), Nuclei (DAPI, blue) in alveoli from *Rap1*$^{fl/fl}$ and *Rap1*^{iECKO} mice at P4.

c, Confocal z-projection images for RAGE (green), isolectin B4 (magenta), and Nuclei (DAPI, blue) in alveoli from *Rap1*$^{fl/fl}$ and *Rap1*^{iECKO} mice at P4 (upper) and P9 (lower).

d, **e**, Cellular localization of YAP in PDGFRα-positive cells isolated from the lungs of *Rap1*$^{fl/fl}$ and *Rap1*^{iECKO} mice at P9. **d**, Confocal fluorescence images for YAP (green), α-SMA (magenta), and Nuclei (DAPI, blue) in PDGFRα-positive cells isolated from the lungs of P9 *Rap1*$^{fl/fl}$ and *Rap1*^{iECKO} mice and cultured on glass-base dishes for 48 h.

e, Quantification of cytoplasmic and nuclear localization of YAP in the PDGFRα-positive cells (arbitrary units, A.U.), as in **d**. Data indicate the average fluorescence intensity of YAP in the cytoplasmic (blue) and nuclear (pink) areas of PDGFRα-positive cells. Data are means ± s.d. (*Rap1*$^{fl/fl}$, n = 114 cells from 12 images; *Rap1*^{iECKO}, n = 85 cells from 18 images).

N.S., no significance; $**P < 0.01$, by one-way ANOVA followed by Tukey's post-hoc test (**e**). Scale bars; 40 μm (**a**), 100 μm (**b**), 20 μm (**c, d**). Source data are provided as a Source data file.

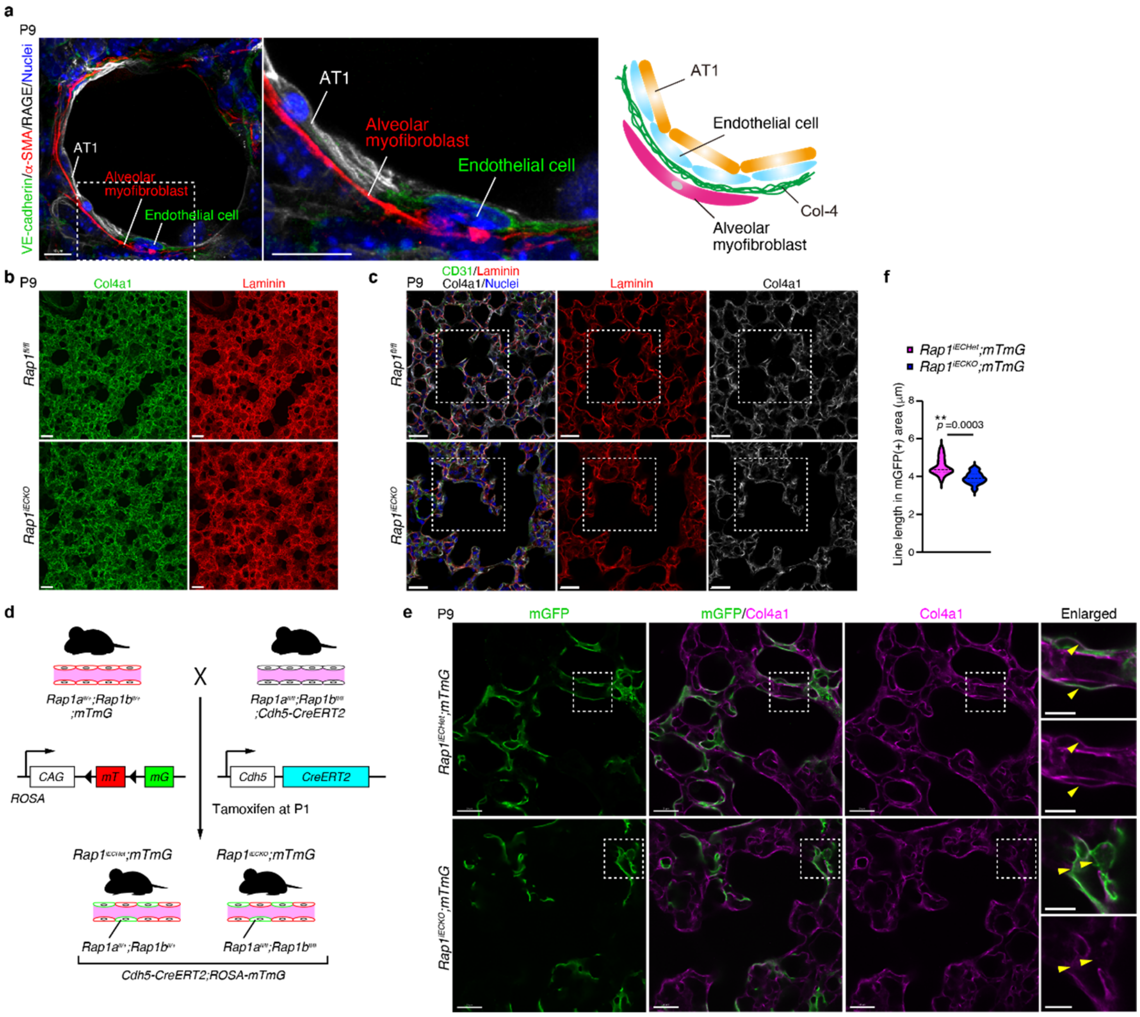

Supplementary Fig. 3. Endothelial Rap1 regulates recruitment of Col-4 into BMs.

a, Representative 10 μm slice images of an alveolus stained with anti-VE-cadherin (green), anti-α-SMA (red), and anti-RAGE (white) antibodies and DAPI (Nuclei, blue) from a *Rap1*$^{fl/fl}$ mouse at P9. Boxed areas are enlarged at the right. The schematic diagram of alveolar structure is shown on the right. Note that capillary ECs surround alveolar type I (AT1) cells to form the alveolus, and myofibroblasts further cover the outside of the alveolus via tight contact with ECs.

b, Confocal z-projection images for Col4a1 (left, green) and Laminin (right, red) in alveoli from *Rap1*$^{fl/fl}$ and *Rap1*^{iECKO} mice at P9.

c, Single slice images for CD31 (green), Laminin (red), Col4a1 (white), and Nuclei (DAPI, blue) in alveoli from *Rap1*$^{fl/fl}$ and *Rap1*^{iECKO} mice at P9. Boxed areas are enlarged in Fig. **3b**.

d, Schematic representation showing the procedure for performing mosaic deletion of *Rap1a* and *Rap1b* in ECs. The detailed procedure is described in the Methods.

e, f, Localization of assembled Col4a1 near control and *Rap1a/b*-deficient alveolar ECs.

e, Single slice images for mGFP (green) and Col4a1 (magenta) alveoli from P9 *Rap1*^{iECHet}*;mTmG* and *Rap1*^{iECKO}*;mTmG* pups that received a low dose of tamoxifen. The mGFP signal indicates control and *Rap1a/b*-deficient alveolar ECs in *Rap1*^{iECHet}*;mTmG* and *Rap1*^{iECKO}*;mTmG* pups, respectively. Boxed areas are enlarged on the right. Note that Col4a1 near *Rap1a/b*-deficient alveolar ECs exhibited reduced assembly, as compared to that in control ECs. **f**, Line lengths of Col4a1 signal in mGFP-positive ECs, as in **e**, were expressed as violin plots (*Rap1*^{iECHet}*;mTmG*; n = 15 images from 3 mice, *Rap1*^{iECKO}*;mTmG*; n = 13 images from 3 mice). Bold and thin dashed lines indicate the median and quartiles, respectively. $^{**}P < 0.01$, by two-tailed Student's *t*-test.
Scale bars; 10 µm (**a** and **e** in enlarged), 50 µm (**b**), 30 µm (**c**), 20 µm (**e**). Source data are provided as a Source data file.

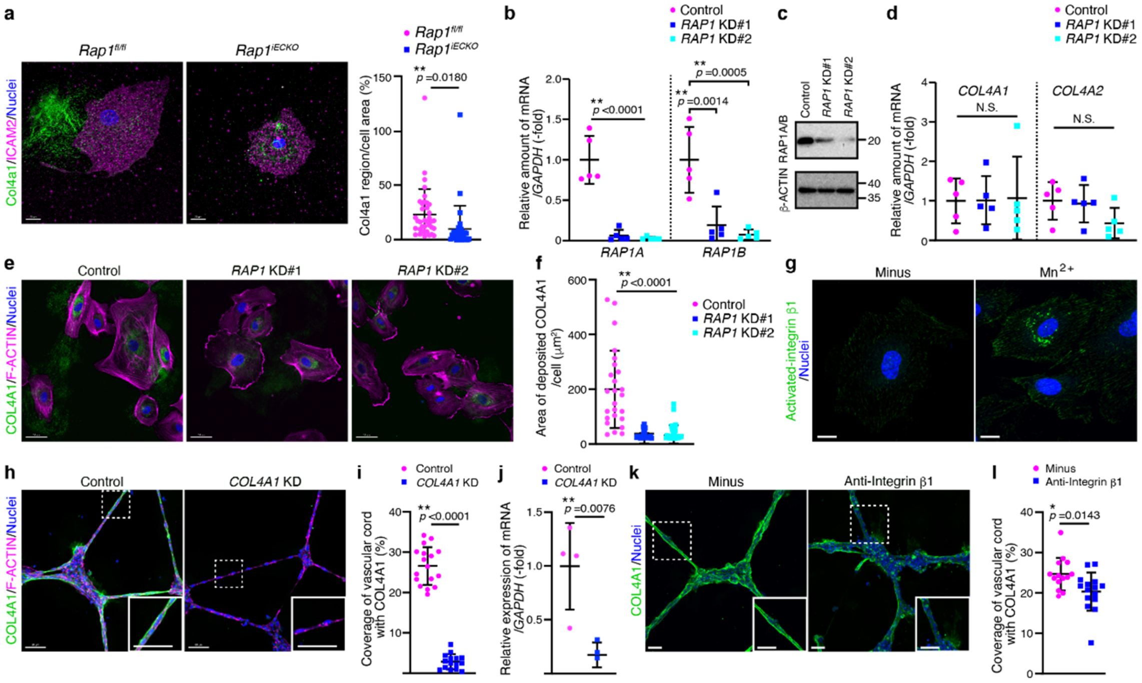

Supplementary Fig. 4. EC-derived Col-4 accumulates around vascular cord structures.

a, Left, confocal fluorescence images for Col4a1 (green), ICAM2 (magenta), and Nuclei (DAPI, bule) in ECs isolated from lungs of P9 *Rap1*$^{fl/fl}$ and *Rap1*^{iECKO} mice and cultured on collagen-coated dishes for at 48 h. The cells were permeabilized prior to antibody staining. Right, the amount of Col4a1 assembled on the dish was quantified as described in Fig. 5**b**. Data are means ± s.d. (*Rap1*$^{fl/fl}$, n = 46 cells from 3 mice; *Rap1*^{iECKO}, n = 41 cells from 3 mice).

b, **c**, Assessment of siRNA-mediated knockdown of *RAP1A* and *RAP1B* in HUVECs by quantitative PCR analysis and Western blot analysis. HUVECs were transfected with either control siRNA or two different sets of siRNA mixtures targeting both *RAP1A* and *RAP1B* (*RAP1* KD#1, *RAP1* KD#2). **b**, Amounts of *RAP1A* (left) and *RAP1B* (right) mRNAs, normalized by that of *GAPDH*, are expressed relative to those in control group (n = 5 experiments for each group). **c**, Western blot analysis of RAP1A/RAP1B (upper) and β-Actin (lower) using the antibody that recognizes both RAP1A and RAP1B and anti-β-Actin antibody.

d, Effects of *RAP1A* and *RAP1B* knockdown on the expression levels of *COL4A1* and *COL4A2* mRNAs. Amounts of *COL4A1* (left) and *COL4A2* (right) mRNAs, normalized by that of *GAPDH*, are expressed relative to those in control group (n = 5 experiments for each group).

e, Confocal fluorescence images for COL4A1 (green), F-actin (magenta), and Nuclei (DAPI, blue) in HUVECs transfected with the indicated siRNAs and cultured on

collagen-coated dishes for 24 h. The cells were permeabilized prior to antibody staining.
f, The amounts of COL4A1 assembled on the dish, as in **e**, are shown as a COL4A1-positive area divided by the number of cells in each image field (control, n = 301 cells from 26 images; *RAP1* KD #1, n = 293 cells from 26 images; *RAP1* KD #2, n = 313 cells from 28 images).
g, Fluorescence images for activated integrin β1 (green) and Nuclei (DAPI, bule) in HUVECs cultured on collagen-coated dishes without (left) and with (right) 0.5 mM $MnCl_2$ for 6 h.
h, Confocal z-projection images for COL4A1 (green), F-actin (magenta), and Nuclei (DAPI, blue) in vascular cord structures constructed by HUVECs transfected with control siRNA or *COL4A1* siRNA and cultured on Matrigel for 36 h. Boxed areas are enlarged in the insets.
i, Percentages of coverage of vascular cord structures with COL4A1, as in **h**, are presented as a percentage relative to the total vascular cord area (n = 17 images/each).
j, Expression levels of *COL4A1* in the HUVECs transfected with control or *COL4A1* siRNA, analyzed by quantitative PCR relative to that of *GAPDH*. (n = 4 experiments/each).
k, Confocal z-projection images for COL4A1 (green) and Nulcei (DAPI, blue) in vascular cord structures constructed by HUVECs cultured on Matrigel in the absence (Minus) and presence (Anti-integrin β1) of blocking antibody against integrin β1 (mAb13). Boxed areas are enlarged in the insets.
l, Percentages of coverage of vascular cord structures with COL4A1, as in **k**, are shown as in **i** (n = 14 images/each).
Each dot represents the result of an individual experiment (**b**, **d**, **j**) and an individual image field (**f**, **i**, **l**), and data are presented as means ± s.d.
N.S., no significance; $^{**}P < 0.01$, $^{*}P < 0.05$, by two-tailed Student's *t*-test (**a, i, j, l**) or one-way ANOVA followed by Tukey's post-hoc test (**b, d, f**). Scale bars; 80 μm (**h**), 50 μm (**e** and **k**), 20 μm (**a** and **g)** and 10 μm (enlarged image in **k)**. Source data are provided as a Source data file.

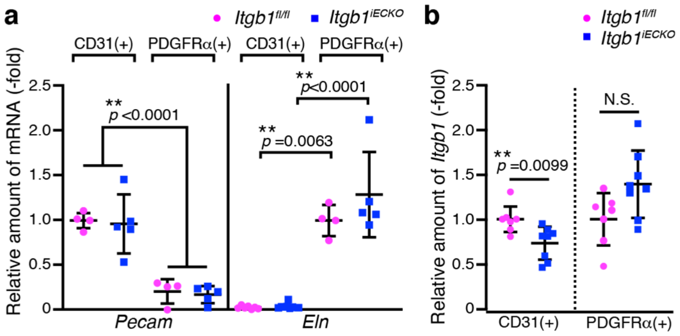

Supplementary Fig. 5. *Itgb1* is partially, but significantly, decreased in ECs isolated from the lungs of *Itgb1*^{iECKO} mice.

a, Expression levels of *Pecam1* (left) and *Eln* (right) in CD31(+) cells and PDGFRα(+) cells isolated from the lungs of *Itgb1*$^{fl/fl}$ and *Itgb1*^{iECKO} mice at P9 were analyzed by quantitative PCR. The values are presented relative to that of *Actb*. Each dot represents an individual mouse. Data are means ± s.d. (*Itgb1*$^{fl/fl}$, n = 4 mice; *Itgb1*^{iECKO}, n = 5 mice).

b, Expression levels of *Itgb1* in CD31(+) cells and PDGFRα(+) cells isolated from the lungs of *Itgb1*$^{fl/fl}$ and *Itgb1*^{iECKO} mice at P9 are shown as in **a**. Data are means ± s.d. (*Itgb1*$^{fl/fl}$, n = 7 mice; *Itgb1*^{iECKO}, n = 8 mice).

N.S., no significance; **$P < 0.01$, by one-way ANOVA followed by Tukey's post-hoc test (**a**) or two-tailed Student's *t*-test (**b**). Source data are provided as a Source data file.

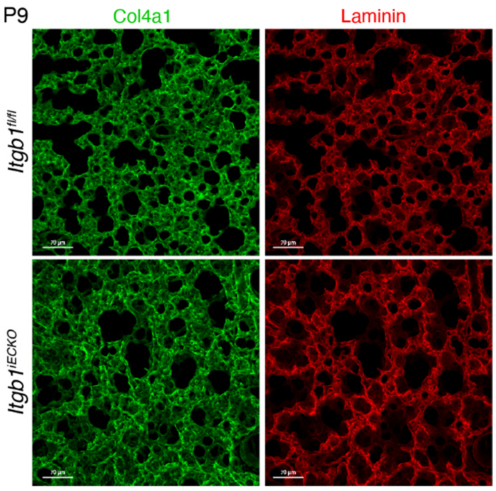

Supplementary Fig. 6. Endothelial integrin β1 generates BMs by recruiting Col-4 and laminin.

Confocal z-projection images for Col4a1 (left, green) and laminin (right, red) in alveoli from *Itgb1*$^{fl/fl}$ and *Itgb1*^{iECKO} mice at P9. Scale bars; 20 µm.

a Quantification of the score for α-SMA bundles

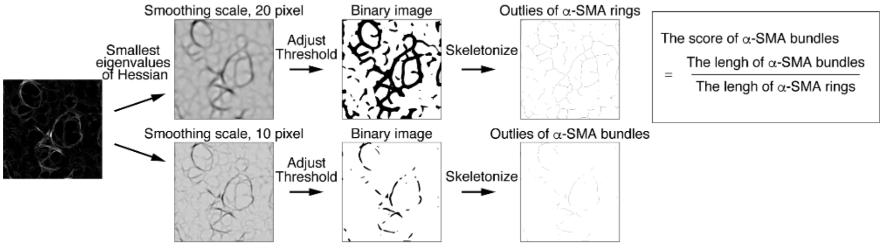

1. Open original 3D image of Cy3-α-SMA. Create Z-projection and convert the image into 8-bit on Fiji software.
2. Compute the smallest eigenvalue of Hessian tensor to extract outlines of alveolar rings, using external plug-in, FeatureJ. Gaussian derivatives (the standard deviation σ= 20 pixels) were calculated in the process.
3. Binarize by adjusting a threshold < -0.16.
4. Execute "skeletonize" and save the image as outlines of α-SMA rings.
5. Using the z-stacked image, compute the smallest eigenvalue of Hessian tensor to extract outlines of α-SMA bundles. Gaussian derivatives (the standard deviation σ= 10 pixels) were calculated in the process.
6. Binarize by adjusting a threshold < -3.0.
7. Execute skeletonize and save the image as Outlines for α-SMA bundles.
8. The length of outline was measured by a manual tracking in the above images.
9. Calculate the values from the length of "α-SMA bundles" divided by the corresponding "α-SMA rings" in each ring.

b Quantification of the cytosolic and nuclear localization of YAP.

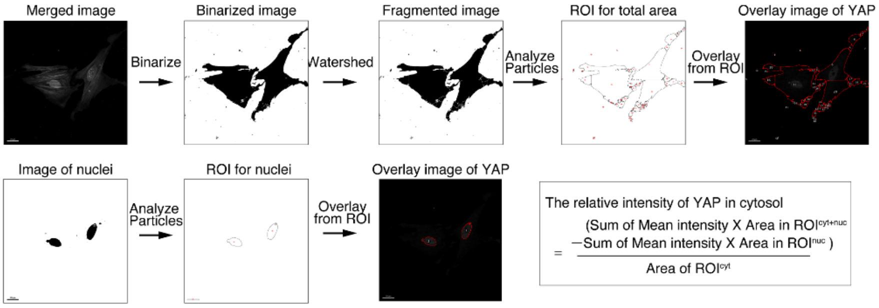

1. Open a merged image on Fiji software, convert scale to 8 bit and adjust the lower limit threshold to 10 .
2. Execute 'Watershed' in the process and run 'Analyze Particles' with the size setting as 2-infinity and add to ROI manager. ($ROI^{cyt+nuc}$)
3. Overlay $ROI^{cyt+nuc}$ on original YAP image, run Measure and calculate the area as well as the intensity of each $ROI^{cyt+nuc}$.
4. Open the corresponding image of nuclei on Fiji software, convert scale to 8 bit and adjust the lower limit of 'Threshold' to 10.
5. Set scale, execute 'Analyze Particles' with the size setting as 10-infinity and add to ROI manager as ROI^{nuc}.
6. Overlay $ROI^{cyt+nuc}$ on original YAP image, run 'Measure' and calculate the area as well as the intensity of each ROI^{nuc}.
7. Calculate total intensity for each $ROI^{cyt+nuc}$ as well as ROI^{nuc}.
8. Estimate total intensity and area of ROI^{cyt} by subtracting total intensity and total area of ROI^{nuc} from $ROI^{cyt+nuc}$.
9. Devide total intensity by total area of ROI^{cyt} as 'Mean intensity' of ROI^{cyt}.

c Quantification of the thickness of Elastin fibers.

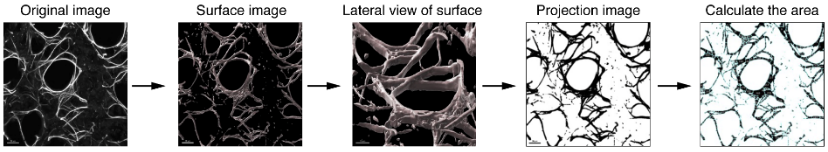

1. Open an original image on Imaris software, make a surface against the staining with Alexa Fluor 633^{TM} Hydrazide.
2. The volumes were calculated automatically on imaris software.
3. Open the same image on Fiji and make projection image.
3. Set scale, adjust threshold of the lower limit to 30, run water shed for the calculation of binalize the image.
4. The segmented image is further processed by the function, 'Analyze particles' and execute 'Measure' to calculate the area.
5. Elastin thickness is calculated as the division from the total volume of surface/the total area on Fiji.

d Quantification of the ratio of activated to total integrin β1 in the plasma membrane of endothelial cells.

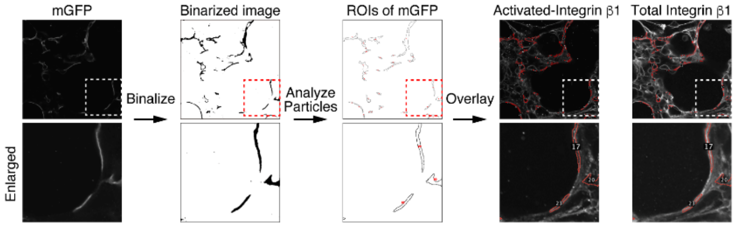

1. Convert the all channels of images into 8 bit scale,
2. Define mGFP-positive area by binarization setting the lower limit as 30.
3. Set scale, run the function, analyze particles and add to ROIs to ROI manager.
(Parameters; size,1.5-Infinity, check, Add to Manager)
4. Overlay ROIs from ROI manager onto the both images from activated-Integrin β1 and total Integrin β1.
5. Measure Mean intensities without modification in each ROI.
6. Calculate the ratio by devision of the mean intensty from activated-Integrin β1 by total Integrin β1 in each ROI.
Lower panels show enlarged images of the boxed area.

Supplementary Fig. 7. Schematic representation of the protocols for data quantification.

a, Protocol to quantify the score of α-SMA bundles in Fig. 2d.

b, Protocol to quantify the cytoplasmic and nuclear localizations of YAP in Fig. 2h and Supplementary Fig. 2e.

c, Protocol to quantify the thickness of Elastin fibers in Fig. 3i.

d, Protocol to quantify the ratio of activated to total Integrin β1 in mGFP-labeled plasma membranes of ECs in Fig. 4g.

Supplemental Tables

Supplementary Table 1. Antibodies and probes used for this study

Primary antibodies for IF

Protein name (clone name)	Company	Product number
AlexaFluorTM647-ERG	Abcam	Ab196149
Carbonic Anhydrase 4	R&D Systems	AF2414
CD29 Rat anti-Mouse activated	BD Biosciences	553715
CD29 Rat anti-Human mAb13	Merk Millipore	MABT821
CD31(Pecam1)	Merk Millipore	MAB1398
	BD Biosciences	550274
	R&D SYSTEMS	AF3628
Col4a1	Merk Millipore	AB769
	Abcam	Ab6586
Cy3-α-SMA	Sigma-Aldrich	C6198
GFP	Thermo Fisher Scientific	A11122
ICAM2/CD102	BD Biosciences	553326
Integrinβ1	R&D Systems	AF2405
Laminin	Sigma-Aldrich	L9393
Neurofilament(NF-M)	Developmental Studies Hybridoma Bank (DSHB)	2H3
Phospho-Myosin Light Chain 2 (Ser19)	Cell Signaling Technology	3671
PLVAP	BD Biosciences	553849
RAGE	R&D Systems	MAB1179
Vincullin (VIN-11-5)	Sigma-Aldrich	SAB4200729
YAP	Novus biologicals	NB110-58358

Probes for IF

Product name	Company	Product number
AlexaFluorTM647-GS-IB4	Thermo Fisher Scientific	I32450
Alexa FluorTM 633-Hydrazide	Thermo Fisher Scientific	10216442
Rhodamine Phalloidin	Thermo Fisher Scientific	R415

Antibody for FACS

Protein name	Company	Product number
FITC anti-mouse TER119/Erythroid Antibody	BioLegend Inc.	116205
FITC anti-mouse CD45 Antibody	BioLegend Inc.	103107
FITC anti-mouse CD326(Ep-CAM) Antibody	BioLegend Inc.	118207
PE anti-mouse CD31 Antibody	BioLegend Inc.	102507
CD140a(PDGFRA) Monoclonal Antibody (APA5), APC, eBioscience™	Thermo Fisher Scientific	17-1401-81

Primary antibody for WB

Protein name (clone name)	Company	Product number
β-actin (Ab-5)	BD Bioscience	612656
Col4a1	Abcam	Ab6586
Col4a1	Merk Millipore	Ab769
YAP	Novus biologicals	NB110-58358

Secondary antibody for WB

Product name	Company	Product number
Anti-Mouse IgG, HRP-Linked Whole Ab Sheep	cytiva	NA931
Anti-Rabbit IgG, HRP-Linked Whole Ab Donkey	cytiva	NA934
HRP-conjugated Affinipure Rabbit Anti-Goat IgG(H+L)	proteintech	SA00001-4

Supplementary Table 2. Primer sequences for qPCR and genotyping and siRNA sequences

qPCR

Gene	F/R	Sequence	Organism
Actb	Forward	GTGACGTTGACATCCGTAAAGA	Mouse
	Reverse	GCCGGACTCATCGTACTCC	
Rap1a	Forward	GCATCATGCGTGAGTACAAG	Mouse
	Reverse	ACCTCGACTTGCTTTCTGTAG	
Rap1b	Forward	GTGAATATAAGCTCGTCGTGC	Mouse
	Reverse	ACACTGCTGTGCATCTACTTC	
Itgb1	Forward	ATGAATTTGCAACTGGTTTCCTG	Mouse
	Reverse	CAGAAGTAGGCATTCCTTCTTGC	
Elastin	Forward	ATGGCGGGTCTGACAGCGGTAG	Mouse
	Reverse	GGTTTTCCTTCCAGGTCCCAGAG	
Pecam1	Forward	CCATGGAAGAAAGGGCTCATTGC	Mouse
	Reverse	CGTAATGGCTGTTGGCTTCCA	
Col4a1	Forward	GCAGAGATGGTCTTGAAGGATTGC	Mouse
	Reverse	AGTTCCTGCTCTTCCTGGCATG	
Col4a2	Forward	GGTTTTCCTGGCCTTGATGGAGA	Mouse
	Reverse	ATGGATGGGGCGAGTAGACAGA	
COL4A1	Forward	GGATGCTGTTGAAAGGTGAAAGA	Human
	Reverse	GGTGGTCCGGAAATCCTGG	
COL4A2	Forward	TTGGCGGGTGTGAAGAAGTTT	Human
	Reverse	CCTTGTCTCCTTTACGTCCCTG	
GAPDH	Forward	ATGGGGAAGGTGAAGGTCG	Human
	Reverse	GGGGTCATTGATGGCAACAATA	

Genotyping

Gene	F/R	Sequence	Organism
Rap1a flox	Forward	GATGGCCAAGGCTCTCAGT	Mouse
	Reverse	CGTATATGAGTGCTTTATCTGCAC	
Rap1b flox	Forward	TGCCCTCTCATGCTATTCCT	Mouse
	Reverse	TTCAAAGTGCGTGCTGTCTC	
Cdh5-CreERT2	Forward	TGCCTATCCTCTTTCCCCAGAT	Mouse
	Reverse	CATTGCTGTCACTTGGTCGTG	
Ingβ flox	Forward	AGG TGC CCT TCC CTC TAG A	Mouse
	Reverse	GTG AAG TAG GTG AAA GGT AAC	

siRNA Target sequence

siRNA name (Ta	Sequence	Organism
#1 RAP1A	GCAAGACAGUGGUGUAACU	Human
#1 RAP1B	GGACAAGGAUUUGCAUUAG	
#2 RAP1A	GCGAGUAGUUGGCAAAGAG	
#2 RAP1B	AAAAUACGAUCCUACGAUA	
COL4A1	CAAAGGUGUUGACGGCUUA	Human
	CGCAAACGCUUACAGCUUU	
	AGAGUUGCCUCAUCUGUGA	
	GGGCAUGCCUGGUAUUGGU	

Supplementary Table 3. Detailed statistics

Figure	Subject	n	Comparison	Significance	Adjusted p value
Fig. 1a	$Rap1^{f/f}$	22 mice			
	$Rap1^{iECKO}$	22 mice			
			$Rap1^{f/f}$ vs $Rap1^{iECKO}$	**	<0.0001
Fig. 1c	$Rap1^{f/f}$	6 mice			
	$Rap1^{iECKO}$	6 mice			
			$Rap1^{f/f}$ vs $Rap1^{iECKO}$	**	0.0026
Fig. 1e	$Rap1^{f/f}$	6 mice			
	$Rap1^{iECKO}$	9 mice			
			$Rap1^{f/f}$ vs $Rap1^{iECKO}$	**	0.0002
Fig. 2b [PDGFRα(+)]	$Rap1^{f/f}$	6 mice			
	$Rap1^{iECKO}$	8 mice			
			$Rap1^{f/f}$ vs $Rap1^{iECKO}$	N.S.	0.4794
Fig. 2b [PDGFRα(+)/α-SMA(+)]	$Rap1^{f/f}$	6 mice			
	$Rap1^{iECKO}$	8 mice			
			$Rap1^{f/f}$ vs $Rap1^{iECKO}$	N.S.	0.7561
Fig. 2b [α-SMA(+)]	$Rap1^{f/f}$	6 mice			
	$Rap1^{iECKO}$	8 mice			
			$Rap1^{f/f}$ vs $Rap1^{iECKO}$	N.S.	0.5908
Fig. 2d	$Rap1^{f/f}$	8 mice			
	$Rap1^{iECKO}$	6 mice			
			$Rap1^{f/f}$ vs $Rap1^{iECKO}$	*	0.0219
Fig. 2f	$Rap1^{f/f}$	5 mice			
	$Rap1^{iECKO}$	5 mice			
			$Rap1^{f/f}$ vs $Rap1^{iECKO}$	**	0.0002
Fig. 2h [α-SMA(+)]	$Rap1^{f/f}$	6 mice			
	$Rap1^{iECKO}$	8 mice			
			$Rap1^{f/f}$ vs $Rap1^{iECKO}$	N.S.	0.2088
Fig. 2h [YAP(+)]	$Rap1^{f/f}$	6 mice			
	$Rap1^{iECKO}$	8 mice			
			$Rap1^{f/f}$ vs $Rap1^{iECKO}$	*	0.0149
Fig. 2b [α-SMA(+)/YAP(+)]	$Rap1^{f/f}$	6 mice			
	$Rap1^{iECKO}$	8 mice			
			$Rap1^{f/f}$ vs $Rap1^{iECKO}$	*	0.0181
Fig. 2j	$Rap1^{f/f}$	6 mice			
	$Rap1^{iECKO}$	5 mice			
			$Rap1^{f/f}$ vs $Rap1^{iECKO}$	*	0.0198
Fig. 2l	$Rap1^{f/f}$	4 mice			
	$Rap1^{iECKO}$	4 mice			
			$Rap1^{f/f}$ vs $Rap1^{iECKO}$	N.S.	0.3581
Fig. 3c (Col4a1)	$Rap1^{f/f}$	4 mice			
	$Rap1^{iECKO}$	4 mice			

			$Rap1^{f/f}$ vs $Rap1^{iECKO}$	**	0.0007
Fig. 3c (Laminin)	$Rap1^{f/f}$	4 mice			
	$Rap1^{iECKO}$	4 mice			
			$Rap1^{f/f}$ vs $Rap1^{iECKO}$	N.S.	0.1741
Fig. 3f	$Rap1^{f/f}$	6 mice			
	$Rap1^{iECKO}$	5 mice			
			$Rap1^{f/f}$ vs $Rap1^{iECKO}$	**	0.0012
Fig. 3g (Col4a1)	$Rap1^{f/f}$	3 mice			
	$Rap1^{iECKO}$	3 mice			
			$Rap1^{f/f}$ vs $Rap1^{iECKO}$	N.S	0.237
Fig. 3g (Col4a2)	$Rap1^{f/f}$	3 mice			
	$Rap1^{iECKO}$	3 mice			
			$Rap1^{f/f}$ vs $Rap1^{iECKO}$	N.S	0.3736
Fig. 3g (Rap1a)	$Rap1^{f/f}$	3 mice			
	$Rap1^{iECKO}$	3 mice			
			$Rap1^{f/f}$ vs $Rap1^{iECKO}$	**	0.0044
Fig. 3g (Rap1b)	$Rap1^{f/f}$	3 mice			
	$Rap1^{iECKO}$	3 mice			
			$Rap1^{f/f}$ vs $Rap1^{iECKO}$	*	0.0305
Fig. 3i	$Rap1^{f/f}$	3 mice			
	$Rap1^{iECKO}$	3 mice			
			$Rap1^{f/f}$ vs $Rap1^{iECKO}$	*	0.022
Fig. 3j	$Rap1^{f/f}$	3 mice			
	$Rap1^{iECKO}$	3 mice			
			$Rap1^{f/f}$ vs $Rap1^{iECKO}$	N.S	0.9553
Fig. 4b	$Rap1^{f/f}$	90 cells/3 mice			
	$Rap1^{iECKO}$	71 cells/3 mice			
			$Rap1^{f/f}$ vs $Rap1^{iECKO}$	**	<0.0001
Fig. 4c (0<x≤5)	$Rap1^{f/f}$	58 cells/3 mice			
	$Rap1^{iECKO}$	42 cells/3 mice			
			$Rap1^{f/f}$ vs $Rap1^{iECKO}$	**	0.0002
Fig. 4c (5<x≤10)	$Rap1^{f/f}$	58 cells/3 mice			
	$Rap1^{iECKO}$	42 cells/3 mice			
			$Rap1^{f/f}$ vs $Rap1^{iECKO}$	**	<0.0001
Fig. 4c (10<x≤20)	$Rap1^{f/f}$	58 cells/3 mice			
	$Rap1^{iECKO}$	42 cells/3 mice			
			$Rap1^{f/f}$ vs $Rap1^{iECKO}$	**	<0.0001
Fig. 4c (20<x)	$Rap1^{f/f}$	58 cells/3 mice			
	$Rap1^{iECKO}$	42 cells/3 mice			
			$Rap1^{f/f}$ vs $Rap1^{iECKO}$	**	<0.0001
Fig. 4e	$Rap1^{f/f}$	32 cells/3 mice			
	$Rap1^{iECKO}$	29 cells/3 mice			
			$Rap1^{f/f}$ vs $Rap1^{iECKO}$	**	<0.0001
Fig. 4g	$Rap1^{iECHet}$;mTmG	460 ROIs/3 mice			

	$Rap1^{iECKO}$;mTmG	191 ROIs/3 mice			
			$Rap1^{iECHet}$;mTmG vs $Rap1^{iECKO}$;mTmG	**	0.0005
Fig. 5b	$Rap1^{f/f}$	44 cells/3 mice			
	$Rap1^{iECKO}$	39 cells/3 mice			
			$Rap1^{f/f}$ vs $Rap1^{iECKO}$	*	0.0109
Fig. 5d (Control)	Minus	125 cells/15 images			
	Mn^{2+}	129 cells/20 images			
			Minus vs Mn^{2+}	N.S.	0.1816
Fig. 5d (Rap1KD#1)	Minus	175 cells/23 images			
	Mn^{2+}	149 cells/20 images			
			Minus vs Mn^{2+}	**	<0.0001
Fig. 5d (Rap1KD#2)	Minus	132 cells/23 images			
	Mn^{2+}	118 cells/20 images			
			Minus vs Mn^{2+}	**	0.0005
Fig. 5f (Conditioned media)	Control	3 independent experiments			
	RAP1KD#1	3 independent experiments			
	RAP1KD#2	3 independent experiments			
			Control vs RAP1KD#1	N.S.	0.8488
			Control vs RAP1KD#2	N.S.	0.9936
			RAP1KD#1 vs RAP1KD#2	N.S.	0.7934
Fig. 5f (Whole cell lysates)	Control	3 independent experiments			
	RAP1KD#1	3 independent experiments			
	RAP1KD#2	3 independent experiments			
			Control vs RAP1KD#1	N.S.	0.9924
			Control vs RAP1KD#2	N.S.	0.9064
			RAP1KD#1 vs RAP1KD#2	N.S.	0.8535
Fig. 5h (Control)	Minus	10 images			
	Mn^{2+}	8 images			
			Minus vs Mn^{2+}	N.S.	0.0946
Fig. 5h (Rap1KD#1)	Minus	11 images			
	Mn^{2+}	11 images			

			Minus vs Mn^{2+}	**	0.0065
Fig. 5h (Rap1KD#2)	Minus	10 images			
	Mn^{2+}	9 images			
			Minus vs Mn^{2+}	**	0.0007
Fig. 6b	$Itgb1^{f/f}$	6 mice			
	$Itgb1^{iECKO}$	6 mice			
			$Itgb1^{f/f}$ vs $Itgb1^{iECKO}$	**	<0.0001
Fig. 6e	$Itgb1^{f/f}$	6 mice			
	$Itgb1^{iECKO}$	6 mice			
			$Itgb1^{f/f}$ vs $Itgb1^{iECKO}$	**	0.0033
Fig. 6g [α-SMA(+)]	$Itgb1^{f/f}$	6 mice			
	$Itgb1^{iECKO}$	6 mice			
			$Itgb1^{f/f}$ vs $Itgb1^{iECKO}$	N.S.	0.1816
Fig. 6g [YAP(+)]	$Itgb1^{f/f}$	6 mice			
	$Itgb1^{iECKO}$	6 mice			
			$Itgb1^{f/f}$ vs $Itgb1^{iECKO}$	**	0.0002
Fig. 6g [α-SMA(+)/YAP(+)]	$Itgb1^{f/f}$	6 mice			
	$Itgb1^{iECKO}$	6 mice			
			$Itgb1^{f/f}$ vs $Itgb1^{iECKO}$	**	0.0067
Fig. 7c (Col4a1)	$Itgb1^{f/f}$	3 mice			
	$Itgb1^{iECKO}$	6 mice			
			$Itgb1^{f/f}$ vs $Itgb1^{iECKO}$	*	0.0346
Fig. 7c (Laminin)	$Itgb1^{f/f}$	3 mice			
	$Itgb1^{iECKO}$	6 mice			
			$Itgb1^{f/f}$ vs $Itgb1^{iECKO}$	N.S.	0.1081
Supple. Fig. 1b (Pecam1)	$Rap1^{f/f}$,CD31(+)	4 mice			
	$Rap1^{iECKO}$,CD31(+)	4 mice			
	$Rap1^{f/f}$,PDGFRα(+)	4 mice			
	$Rap1^{iECKO}$,PDGFRα(+)	4 mice			
			$Rap1^{f/f}$,CD31(+) vs $Rap1^{f/f}$,PDGFRα(+)	**	<0.0001
			$Rap1^{f/f}$,CD31(+) vs $Rap1^{iECKO}$,PDGFRα(+)	**	<0.0001
			$Rap1^{iECKO}$,CD31(+) vs $Rap1^{f/f}$,PDGFRα(+)	**	<0.0001
			$Rap1^{iECKO}$,CD31(+) vs $Rap1^{iECKO}$,PDGFRα(+)	**	<0.0001
Supple. Fig. 1b (Eln)	$Rap1^{f/f}$,CD31(+)	4 mice			
	$Rap1^{iECKO}$,CD31(+)	4 mice			
	$Rap1^{f/f}$,PDGFRα(+)	4 mice			
	$Rap1^{iECKO}$,PDGFRα(+)	4 mice			
			$Rap1^{f/f}$,CD31(+) vs $Rap1^{f/f}$,PDGFRα(+)	**	0.001
			$Rap1^{f/f}$,CD31(+) vs $Rap1^{iECKO}$,PDGFRα(+)	**	<0.0001

			Rap1^{iECKO},CD31(+) vs Rap1$^{f/f}$,PDGFRα(+)	**	0.0017
			Rap1^{iECKO},CD31(+) vs Rap1^{iECKO},PDGFRα(+)	**	<0.0001
Supple. Fig. 1c (Rap1a)	Rap1$^{f/f}$,CD31(+)	4 mice			
	Rap1^{iECKO},CD31(+)	4 mice			
	Rap1$^{f/f}$,PDGFRα(+)	4 mice			
	Rap1^{iECKO},PDGFRα(+)	4 mice			
			Rap1$^{f/f}$,CD31(+) vs Rap1^{iECKO},CD31(+)	**	<0.0001
			Rap1$^{f/f}$,PDGFRα(+) vs Rap1^{iECKO},PDGFRα(+)	N.S.	>0.9999
Supple. Fig. 1c (Rap1b)	Rap1$^{f/f}$,CD31(+)	4 mice			
	Rap1^{iECKO},CD31(+)	4 mice			
	Rap1$^{f/f}$,PDGFRα(+)	4 mice			
	Rap1^{iECKO},PDGFRα(+)	4 mice			
			Rap1$^{f/f}$,CD31(+) vs Rap1^{iECKO},CD31(+)	**	<0.0001
			Rap1$^{f/f}$,PDGFRα(+) vs Rap1^{iECKO},PDGFRα(+)	N.S.	>0.9999
Supple. Fig. 1e	Rap1$^{f/f}$	7 mice			
	Rap1^{iECKO}	7 mice			
			Day 0: Rap1$^{f/f}$ vs Rap1^{iECKO}	N.S.	0.2922
			Day 4: Rap1$^{f/f}$ vs Rap1^{iECKO}	N.S.	0.8724
			Day 8: Rap1$^{f/f}$ vs Rap1^{iECKO}	N.S.	0.4266
			Day 14: Rap1$^{f/f}$ vs Rap1^{iECKO}	N.S.	0.2148
Supple. Fig. 1j	Rap1$^{f/f}$	5 mice			
	Rap1^{iECKO}	5 mice			
			Rap1$^{f/f}$ vs Rap1^{iECKO}	N.S.	0.7860
Supple. Fig. 1l	Rap1$^{f/f}$	5 mice			
	Rap1^{iECKO}	5 mice			
			Rap1$^{f/f}$ vs Rap1^{iECKO}	N.S.	0.9178
Supple. Fig. 1m	Rap1$^{f/f}$	5 mice			
	Rap1^{iECKO}	5 mice			
			Rap1$^{f/f}$ vs Rap1^{iECKO}	N.S.	0.2305
Supple. Fig. 2e	Rap1$^{f/f}$	114 cells/12 images			
	Rap1^{iECKO}	85 cells/18 images			
			Rap1$^{f/f}$/Nucleus vs Rap1$^{f/f}$/Cytoplasm	**	<0.0001
			Rap1^{iECKO}/Nucleus vs Rap1^{iECKO}/Cytoplasm	**	<0.0001
			Rap1$^{f/f}$/Nucleus vs Rap1^{iECKO}/Nucleus	N.S.	0.2651

			$Rap1^{f/f}$/Cytoplasm vs $Rap1^{iECKO}$/Cytoplasm	N.S.	0.6525
Supple. Fig. 3f	$Rap1^{iECHet}$;mTmG	15 slices/3 mice			
	$Rap1^{iECKO}$;mTmG	13 slices/3 mice			
			$Rap1^{iECHet}$;mTmG vs $Rap1^{iECKO}$;mTmG	**	0.0003
Supple. Fig. 4a	$Rap1^{f/f}$	46 cells/3 mice			
	$Rap1^{iECKO}$	41 cells/3 mice			
			$Rap1^{f/f}$ vs $Rap1^{iECKO}$	*	0.0180
Supple. Fig. 4b (RAP1A)	Control	5 independent experiments			
	RAP1KD#1	5 independent experiments			
	RAP1KD#2	5 independent experiments			
			Control vs RAP1KD#1	**	<0.0001
			Control vs RAP1KD#2	**	<0.0001
Supple. Fig. 4b (RAP1B)	Control	5 independent experiments			
	RAP1KD#1	5 independent experiments			
	RAP1KD#2	5 independent experiments			
			Control vs RAP1KD#1	**	0.0014
			Control vs RAP1KD#2	**	0.0005
Supple. Fig 4d (COL4A1)	Control	5 independent experiments			
	RAP1KD#1	5 independent experiments			
	RAP1KD#2	5 independent experiments			
			Control vs RAP1KD#1	N.S.	0.9994
			Control vs RAP1KD#2	N.S.	0.9885
Supple. Fig 4d (COL4A2)	Control	5 independent experiments			
	RAP1KD#1	5 independent experiments			
	RAP1KD#2	5 independent experiments			
			Control vs RAP1KD#1	N.S.	0.9682
			Control vs RAP1KD#2	N.S.	0.1562
Supple. Fig. 4f	Control	301 cells/26 images			
	RAP1KD#1	293 cells/26 images			

	RAP1KD#2	313 cells/28 images			
			Control vs RAP1KD#1	**	<0.0001
			Control vs RAP1KD#2	**	<0.0001
Supple. Fig. 4i	Control	17 images			
	COL4A1 KD	17 images			
			Control vs COL4A1 KD	**	<0.0001
Supple. Fig. 4j	Control	4 independent experiments			
	COL4A1 KD	4 independent experiments			
			Control vs COL4A1 KD	**	0.0076
Supple. Fig. 4l	Control	14 images			
	Anti-integrin β1	14 images			
			Control vs Anti-integrin β1	*	0.0143
Supple. Fig. 5a (Pecam1)	Itgb1$^{f/f}$,CD31(+)	4 mice			
	Itgb1^{iECKO},CD31(+)	5 mice			
	Itgb1$^{f/f}$,PDGFRα(+)	4 mice			
	Itgb1^{iECKO},PDGFRα(+)	5 mice			
			Itgb1$^{f/f}$,CD31(+) vs Itgb1$^{f/f}$,PDGFRα(+)	**	<0.0001
			Itgb1$^{f/f}$,CD31(+) vs Itgb1^{iECKO},PDGFRα(+)	**	<0.0001
			Itgb1^{iECKO},CD31(+) vs Itgb1$^{f/f}$,PDGFRα(+)	**	<0.0001
			Itgb1^{iECKO},CD31(+) vs Itgb1^{iECKO},PDGFRα(+)	**	<0.0001
Supple. Fig. 5a (Eln)	Itgb1$^{f/f}$,CD31(+)	4 mice			
	Itgb1^{iECKO},CD31(+)	5 mice			
	Itgb1$^{f/f}$,PDGFRα(+)	4 mice			
	Itgb1^{iECKO},PDGFRα(+)	5 mice			
			Itgb1$^{f/f}$,CD31(+) vs Itgb1$^{f/f}$,PDGFRα(+)	**	0.0063
			Itgb1$^{f/f}$,CD31(+) vs Itgb1^{iECKO},PDGFRα(+)	**	0.0002
			Itgb1^{iECKO},CD31(+) vs Itgb1$^{f/f}$,PDGFRα(+)	**	0.0029
			Itgb1^{iECKO},CD31(+) vs Itgb1^{iECKO},PDGFRα(+)	**	<0.0001
Supple. Fig. 5b	Itgb1$^{f/f}$,CD31(+)	7 mice			
	Itgb1^{iECKO},CD31(+)	8 mice			
	Itgb1$^{f/f}$,PDGFRα(+)	7 mice			
	Itgb1^{iECKO},PDGFRα(+)	8 mice			
			Itgb1$^{f/f}$,CD31(+) vs Itgb1^{iECKO},CD31(+)	**	0.0099

			$Itgb1^{f/f}$,PDGFRα(+) vs $Itgb1^{iECKO}$,PDGFRα(+)	N.S.	0.1079

Data were considered statistically significant if the p value was less than 0.05. No significant difference, $p < 0.05$ and $p < 0.01$ are shown as N.S., *, and **, respectively.
