# Supplementary material for: Endothelial cells regulate alveolar morphogenesis by constructing basement membranes acting as a scaffold for myofibroblasts
Source: Nat Commun. 2024 Mar 4;15:1622. doi: 10.1038/s41467-024-45910-y (PMC10912381; doi:10.1038/s41467-024-45910-y)
Supplement: Supplementary file 3 — Description of Additional Supplementary Files [file 41467_2024_45910_MOESM3_ESM.pdf]

Description of Additional Supplementary Files

File Name: Supplementary Movie 1
Description: 3D confocal image of anti-α-SMA antibody-stained alveoli in the lung of control mouse. See also Fig. 2c.

File Name: Supplementary Movie 2
Description: 3D confocal image of anti-α-SMA antibody-stained alveoli in the lung of *Rap1*^{iECKO} mouse. Note that myofibroblasts in *Rap1*^{iECKO} mice had thinner α-SMA filaments than control myofibroblasts (Supplementary Movie 1). See also Fig. 2c.

File Name: Supplementary Movie 3
Description: 3D confocal image of Alexa Fluor 633-Hydrazide-stained Elastin fibers in the lung of control mouse. See also Fig. 3h.

File Name: Supplementary Movie 4
Description: 3D confocal image of Alexa Fluor 633-Hydrazide-stained Elastin fibers in the lung of *Rap1*^{iECKO} mouse. Note that myofibroblasts in *Rap1*^{iECKO} mice had thinner Elastin fiber than control myofibroblasts (Supplementary Movie 3). See also Fig. 3h.
