# Supplementary material for: Endothelial cells regulate alveolar morphogenesis by constructing basement membranes acting as a scaffold for myofibroblasts
Source: Nat Commun. 2024 Mar 4;15:1622. doi: 10.1038/s41467-024-45910-y (PMC10912381; doi:10.1038/s41467-024-45910-y)
Supplement: Supplementary file 8 — Reporting Summary [file 41467_2024_45910_MOESM8_ESM.pdf]

nature portfolio

Corresponding author(s): Shigetomo Fukuhara
Haruko Watanabe-Takano

Last updated by author(s): Dec 22, 2023

Reporting Summary

Nature Portfolio wishes to improve the reproducibility of the work that we publish. This form provides structure for consistency and transparency in reporting. For further information on Nature Portfolio policies, see our Editorial Policies and the Editorial Policy Checklist.

Statistics

For all statistical analyses, confirm that the following items are present in the figure legend, table legend, main text, or Methods section.

n/a	Confirmed	
☐	☒	The exact sample size (n) for each experimental group/condition, given as a discrete number and unit of measurement
☐	☒	A statement on whether measurements were taken from distinct samples or whether the same sample was measured repeatedly
☐	☒	The statistical test(s) used AND whether they are one- or two-sided<br>*Only common tests should be described solely by name; describe more complex techniques in the Methods section.*
☒	☐	A description of all covariates tested
☐	☒	A description of any assumptions or corrections, such as tests of normality and adjustment for multiple comparisons
☐	☒	A full description of the statistical parameters including central tendency (e.g. means) or other basic estimates (e.g. regression coefficient) AND variation (e.g. standard deviation) or associated estimates of uncertainty (e.g. confidence intervals)
☐	☒	For null hypothesis testing, the test statistic (e.g. F, t, r) with confidence intervals, effect sizes, degrees of freedom and P value noted<br>*Give P values as exact values whenever suitable.*
☒	☐	For Bayesian analysis, information on the choice of priors and Markov chain Monte Carlo settings
☒	☐	For hierarchical and complex designs, identification of the appropriate level for tests and full reporting of outcomes
☒	☐	Estimates of effect sizes (e.g. Cohen's d, Pearson's r), indicating how they were calculated

Our web collection on statistics for biologists contains articles on many of the points above.

Software and code

Policy information about availability of computer code

Data collection

Image acquisition for 3D immunofluorescence stained alveoli: Fluorescence images were acquired using a FLUOVIEW FV3000 microscope (Olympus) equipped with an ×20 water objective lens (XLUMPLFLN) (Olympus) or a ×60 silicone objective lens (UPLSAPOXS2) (Olympus) operated with FLUOVIEW FV31S-SW software (Olympus), as described in detail in the Methods section.

Image acquisition for PDGFRα-positive cells, CD31-positive endothelial cells isolated from the lungs of control and Rap1iECKO mice, and human umbilical vein endothelial cells (HUVECs) plated on glass-based dishes: Fluorescence images were acquired using a FLUOVIEW FV1200 confocal inverted microscope with 40× objective lens (UPlanSApo 40x/0.95na Objective, Olympus) or 60× oil objective lens (UPlanSAPO 60x/1.35na oil Objective) and GaAsP photomultiplier tubes operated with FLUOVIEW Ver. 4.2c software (Olympus), as described in detail in the Methods section.

Image acquisition for in vitro cord-like structures constructed by HUVECs on Matrigel: Fluorescence images were acquired employing a FV3000 or FV1200 confocal upright microscope equipped with a 20× water objective lens (XLUMPLFLN) and GaAsP photomultiplier tubes and operated with FLUOVIEW FV31S-SW software, as described in detail in the Methods section.

Image acquisition for HE-stained tissues: Images were obtained employing fluorescence microscopy (KEYENCE, BZ-X710).

Bright field images of collagen gels were acquired with a SZX16 stereomicroscope (Olympus) equipped with a digital camera (Olympus).

Data collection for quantitative PCR analysis: CFX96 Touch Deep Well real-time PCR detection system (Bio-Rad).

Data collection for western blot analysis: Chemiluminescence signals were obtained using the AmershamTM ImageQuantTM 800 (IQ800) or ChemiDoc Tough MP Imaging system (Bio-Rad).

Data analysis

Image data of 3D immunofluorescence stained alveoli, sizes and numbers of cell and focal adhesions/focal complexes in endothelial cells plated on the glass-base dishes, and percentage of α-SMA-positive cells, nuclear YAP-positive cells, and α-SMA-positive cells exhibiting the nuclear YAP signal were analyzed using Imaris 10.0.0 software (Bitplane).

Image data obtained from collagen gel contraction assay, cellular localization of YAP in PDFGRα-positive cells on glass-base dishes, deposited areas of Col4a1 on the glass-base dishes, coverage ratios of endothelial tubes with COL4A1 obtained from Matrigel-based in vitro cord formation assay, fluorescence intensity of pMLC(Ser19) in myofibroblasts in the alveoli, connectivity of Col4a1 and Laminin in the alveoli, score of α-SMA bundles in the avleoli, and ratio of activated to total integrin β1 in the plasma membrane of lung endothelial cells were analyzed using Fiji Version 2.14.0 software (NIH).

Thickness of elastin fibers in the alveoli was analyzed using Imaris 10.0.0 software (Bitplane) and Fiji Version 2.14.0 software (NIH). Densitometry data generated for Western blot analysis was analyzed using AmershamTM ImageQuantTM 800 (IQ800) or ChemiDoc Tough MP Imaging system (Bio-Rad).

Statistical analyses were carried out using GraphPad Prism 10 10.0.2 (GraphPad software, Inc).

For manuscripts utilizing custom algorithms or software that are central to the research but not yet described in published literature, software must be made available to editors and reviewers. We strongly encourage code deposition in a community repository (e.g. GitHub). See the Nature Portfolio guidelines for submitting code & software for further information.

Data

Policy information about availability of data

All manuscripts must include a data availability statement. This statement should provide the following information, where applicable:

- Accession codes, unique identifiers, or web links for publicly available datasets
- A description of any restrictions on data availability
- For clinical datasets or third party data, please ensure that the statement adheres to our policy

The authors declare that all data supporting the findings of this study are available within the main text and supplementary materials. Raw data to generate all graphs within the Figures and Supplementary Figures and uncropped scans of all blots and gels in the Supplementary Figures are provided as a Source Data File. Source data are provided with this paper.

Research involving human participants, their data, or biological material

Policy information about studies with human participants or human data. See also policy information about sex, gender (identity/presentation), and sexual orientation and race, ethnicity and racism.

Reporting on sex and gender	Human participants and human data are not involved in this study.
Reporting on race, ethnicity, or other socially relevant groupings	Human participants and human data are not involved in this study.
Population characteristics	Human participants and human data are not involved in this study.
Recruitment	Human participants and human data are not involved in this study.
Ethics oversight	Human participants and human data are not involved in this study.

Note that full information on the approval of the study protocol must also be provided in the manuscript.

Field-specific reporting

Please select the one below that is the best fit for your research. If you are not sure, read the appropriate sections before making your selection.

☒ Life sciences ☐ Behavioural & social sciences ☐ Ecological, evolutionary & environmental sciences

For a reference copy of the document with all sections, see nature.com/documents/nr-reporting-summary-flat.pdf

Life sciences study design

All studies must disclose on these points even when the disclosure is negative.

Sample size	No statistical method was used to predetermine sample size. Sample size was determined based on previous published studies using similar methodologies in this field (Kato et al. Nat. Commun. 9: 2448, 2018; Li et al. J. Clin. Invest. 130: 2859-2871, 2020). All sample sizes are listed in the corresponding figure legends or on the figures.

Data exclusions	No data were excluded from from the analyses.
Replication	At least 3 independent experimental replicates were performed to confirm the reproducibility of the findings. All replication attempts were successful.
Randomization	After genotyping, control and mutant mice were randomly assigned for the experiments. To acquire confocal fluorescence images, the imaging regions were randomly selected. For in vitro studies, the cells were randomized into different groups prior to treatment.
Blinding	Blinding during group allocation and data collection was not performed since the same investigator did all experimental processes from group allocation to data analysis.

Reporting for specific materials, systems and methods

We require information from authors about some types of materials, experimental systems and methods used in many studies. Here, indicate whether each material, system or method listed is relevant to your study. If you are not sure if a list item applies to your research, read the appropriate section before selecting a response.

Materials & experimental systems

n/a	Involved in the study	
☐	☒	Antibodies
☐	☒	Eukaryotic cell lines
☒	☐	Palaeontology and archaeology
☐	☒	Animals and other organisms
☒	☐	Clinical data
☒	☐	Dual use research of concern
☒	☐	Plants

Methods

n/a	Involved in the study	
☒	☐	ChIP-seq
☐	☒	Flow cytometry
☒	☐	MRI-based neuroimaging

Antibodies

Antibodies used

Primary antibodies
Alexa Fluor 647 Rabbit anti-ERG (Abcam, #ab196149, 1:200)
Goat anti-mouse Carbonic Anhydrase 4 (R&D Systems, #AF2414, 1:200)
Rat Anti-Mouse CD29 (BD Biosciences, #553715, 1:200)
Rat anti-Integrin beta1 (CD29) clone mAb13 (Merk Millipore, #MABT821, 1:200)
Hamster anti-PECAM-1 Antibody, clone 2H8 (Merk Millipore, #MAB1398, 1:200)
Rat anti-mouse CD31 clone MEC 13.3 (BD Biosciences, #550274, 1:200)
Goat anti-human/mouse/rat CD31/PECAM-1 (R&D Systems, #AF2414, 1:200)
Goat anti-Collagen Type IV (Merk Millipore, #AB769, 1:400)
Rabbit anti-Collagen IV (Abcam, #ab6586, 1:200)
Mouse anti-Actin, α-Smooth Muscle - Cy3 (Sigma-Aldrich, #C6198, 1:200)
Rabbit anti-GFP (Thermo Fisher Scientific, #A11122, 1:400)
Rat anti-mouse CD102 (BD Biosciences, #553326, 1:200)
Goat mouse/rat Integrin beta 1/CD29 (R&D Systems, #AF2405, 1:200)
Rabbit anti-Laminin (Sigma-Aldrich, #L9393, 1:400)
Mouse anti-Neurofilament (NF-M) (Developmental Studies Hybridoma Bank, #2H3, 1:200)
Rabbit anti-Phospho-Myosin Light Chain 2 (Ser19) Antibody (Cell Signaling Technology, #3671, 1:200)
Rat Anti-Mouse Panendothelial Cell Antigen/Plvap (BD Biosciences, #553849, 1:200)
Rat anti-Mouse/Rat RAGE (R&D Systems, #MAB1179, 1:200)
Mouse Anti-Vinculin clone VIN-11-5 (Sigma-Aldrich, #SAB4200729, 1:400)
Rabbit anti-YAP1 (Novus biologicals, #NB110-58358, 1:200)
FITC anti-mouse TER119/Erythroid (BioLegend Inc., #116205, 1:100)
FITC anti-mouse CD45 (BioLegend Inc., #103107, 1:100)
FITC anti-mouse CD326(Ep-CAM) (BioLegend Inc., #118207, 1:100)
PE anti-mouse CD31 (BioLegend Inc., #102507, 1:50)
Anti-CD140a(PDGFRA) (APA5), APC (Thermo Fisher Scientific, #17-1401-81, 1:100)
Anti-Actin Ab-5 (BD Biosciences, #612656, 1:2000)
Mouse IgG HRP Linked Whole Ab (Cytiva, #NA931, 1:5000)
Rabbit IgG HRP Linked Whole Ab(Cytiva, #NA934, 1:5000)
HRP-conjugated Affinipure Rabbit Anti-Goat IgG(H+L) (proteintech, #SA00001-4, 1:5000)

Validation

All the antibodies used in this study were commercially available and validated by manufacturers as described on the following web sites or by previous reports.

Alexa Fluor 647 Rabbit anti-ERG (Abcam, #ab196149):https://www.abcam.com/products/primary-antibodies/alexa-fluor-647-erg-antibody-epr3864-ab196149.html

Goat anti-mouse Carbonic Anhydrase 4 (R&D Systems, #AF2414): https://www.rndsystems.com/products/mouse-carbonic-anhydrase-iv-ca4-antibody_af2414

Rat Anti-Mouse CD29 (BD Biosciences, #553715): https://www.bdbiosciences.com/en-us/products/reagents/flow-cytometry-reagents/research-reagents/single-color-antibodies-ruo/purified-rat-anti-mouse-cd29.553715

Rat anti-Integrin beta1 (CD29) clone mAb13 (Merk Millipore, #MABT821): https://www.emdmillipore.com/US/en/product/Anti-Integrin-beta1-CD29-Antibody-clone-mAb13,MM_NF-MABT821?bd=1

Hamster anti-PECAM-1 Antibody, clone 2H8 (Merk Millipore, #MAB1398): https://www.emdmillipore.com/US/en/product/Anti-PECAM-1-Antibody-clone-2H8-Azide-Free,MM_NF-MAB1398Z#anchor_Product%20Information

Rat Anti-Mouse CD31 clone MEC 13.3 (BD Biosciences, #550274): https://www.bdbiosciences.com/en-us/products/reagents/flow-cytometry-reagents/research-reagents/single-color-antibodies-ruo/purified-rat-anti-mouse-cd31.550274

Goat anti-human/mouse/rat CD31/PECAM-1 (R&D Systems, #AF2414): https://www.rndsystems.com/products/human-mouse-rat-cd31-pecam-1-antibody_af3628

Goat anti-Collagen Type IV (Merk Millipore, #AB769): https://www.emdmillipore.com/US/en/product/Anti-Collagen-Type-IV-Antibody,MM_NF-AB769

Rabbit anti-Collagen IV (Abcam, #abAb6586): https://www.abcam.com/products/primary-antibodies/collagen-iv-antibody-ab6586.html

Mouse anti-Actin, α-Smooth Muscle - Cy3 (Sigma-Aldrich, #C6198): https://www.sigmaaldrich.com/US/en/product/sigma/c6198

Rabbit anti-GFP (Thermo Fisher Scientific, #A11122): https://www.thermofisher.com/antibody/product/GFP-Antibody-Polyclonal/A-11122

Rat anti-mouse CD102 (BD Biosciences, #553326): https://www.bdbiosciences.com/en-us/products/reagents/flow-cytometry-reagents/research-reagents/single-color-antibodies-ruo/purified-rat-anti-mouse-cd102.553326

Goat mouse/rat Integrin beta 1/CD29 (R&D Systems, #AF2405): https://www.rndsystems.com/products/mouse-rat-integrin-beta1-cd29-antibody_af2405

Rabbit anti-Laminin (Sigma-Aldrich, #L9393): https://www.sigmaaldrich.com/US/en/product/sigma/l9393

Mouse anti-Neurofilament (NF-M) (Developmental Studies Hybridoma Bank, #2H3): https://dshb.biology.uiowa.edu/2H3

Rabbit anti-Phospho-Myosin Light Chain 2 (Ser19) Antibody (Cell Signaling Technology, #3671): https://www.cellsignal.jp/products/primary-antibodies/phospho-myosin-light-chain-2-ser19-antibody/3671

Rat Anti-Mouse Panendothelial Cell Antigen/Plvap (BD Biosciences, #553849): https://www.bdbiosciences.com/en-us/products/reagents/western-blotting-and-molecular-reagents/western-blot-reagents/purified-rat-anti-mouse-panendothelial-cell-antigen.553849

Rat anti-Mouse/Rat RAGE (R&D Systems, #MAB1179): https://www.rndsystems.com/products/mouse-rat-rage-antibody-175410_mab1179

Mouse Anti-Vinculin clone VIN-11-5 (Sigma-Aldrich, #SAB4200729): https://www.sigmaaldrich.com/US/en/product/sigma/sab4200729

Rabbit anti-YAP1 (Novus biologicals, #NB110-58358): https://www.novusbio.com/products/yap1-antibody_nb110-58358

FITC anti-mouse TER119/Erythroid (BioLegend Inc., #116205): https://www.biolegend.com/ja-jp/products/fitc-anti-mouse-ter-119-erythroid-cells-antibody-1865

FITC anti-mouse CD45 (BioLegend Inc., #103107): https://www.biolegend.com/ja-jp/products/fitc-anti-mouse-cd45-antibody-99

FITC anti-mouse CD326(Ep-CAM) (BioLegend Inc., #118207): https://www.biolegend.com/ja-jp/products/fitc-anti-mouse-cd326-ep-cam-antibody-4971

PE anti-mouse CD31 (BioLegend Inc., #102507): https://www.biolegend.com/ja-jp/products/pe-anti-mouse-cd31-antibody-379

Anti-CD140a(PDGFRA) (APA5), APC (Thermo Fisher Scientific, #17-1401-81): https://www.thermofisher.com/antibody/product/CD140a-PDGFRA-Antibody-clone-APA5-Monoclonal/17-1401-81

Anti-Actin Ab-5 (BD Biosciences, #612656): https://www.bdbiosciences.com/en-ca/products/reagents/microscopy-imaging-reagents/immunofluorescence-reagents/purified-mouse-anti-actin-ab-5.612657

Mouse IgG HRP Linked Whole Ab (Cytiva, #NA931): https://www.sigmaaldrich.com/US/en/product/sigma/gena9311ml

Rabbit IgG HRP Linked Whole Ab(Cytiva, #NA934): https://www.sigmaaldrich.com/US/en/product/sigma/gena934100ul

HRP-conjugated Affinipure Rabbit Anti-Goat IgG(H+L) (proteintech, #SA00001-4): https://www.ptglab.co.jp/products/HRP-conjugated-Affinipure-Rabbit-Anti-Goat-IgG-H-L-secondary-antibody.htm

Eukaryotic cell lines

Policy information about cell lines and Sex and Gender in Research

Cell line source(s)	Human umbilical vein endothelial cells (HUVECs) were obtained from KURABO (Osaka, Japan).
Authentication	Immunofluorescence staining was performed for Factor VIII-related antigen (positive) and α-actin (negative) to authenticate HUVECs by a manufacturer. Prior to experiments, investigators verified the cell morphology to authenticate HUVECs.
Mycoplasma contamination	HUVECs were tested mycoplasma-negative by the manufacturer.
Commonly misidentified lines (See ICLAC register)	No commonly misidentified cell lines were used in this study.

Animals and other research organisms

Policy information about studies involving animals; ARRIVE guidelines recommended for reporting animal research, and Sex and Gender in Research

Laboratory animals	Mice were housed in 12:12 light:dark light cycles at ambient temperature ranging between 20°C and 23°C and humidities between 30% and 70%. Rap1a and Rap1b double-floxed mice (B6;129S-Rap1atmMorz;Rap1btm1Morz/J), mTmG reporter mice [mTGt(ROSA)26Sortm4(ACTB-tdTomato,-EGFP)Luo /J], Itgb1 floxed mice, and Cdh5-CreERT2 mice in the C57BL/6 background were used in this study. Postnatal mice at P4, P9, P14, and P21 were used for conducting 3D immunofluorescence analyses, HE-staining analysis, and cell isolations. Survival curves of mice were determined up to P31. Body weights of mice were analyzed until P14.
Wild animals	No wild animals were used in this study.
Reporting on sex	Male and female postnatal mice were randomly selected for this study.
Field-collected samples	No field-collected samples were used in this study.
Ethics oversight	Animal experiments were approved by the animal committees of the National Cerebral and Cardiovascular Center, the Nippon Medical School, and Tokyo Medical and Dental University and performed by following the guidelines of the National Cerebral, Cardiovascular Center and the Nippon Medical School, and Tokyo Medical and Dental University.

Note that full information on the approval of the study protocol must also be provided in the manuscript.

Plants

Seed stocks	N/A
Novel plant genotypes	N/A
Authentication	N/A

Flow Cytometry

Plots

Confirm that:

☒ The axis labels state the marker and fluorochrome used (e.g. CD4-FITC).

☒ The axis scales are clearly visible. Include numbers along axes only for bottom left plot of group (a 'group' is an analysis of identical markers).

☒ All plots are contour plots with outliers or pseudocolor plots.

☒ A numerical value for number of cells or percentage (with statistics) is provided.

Methodology

Sample preparation	Cells were isolated from the mouse lungs, resuspended in FACS buffer, and then sorted using cell sorting.
Instrument	BD FACSAriaTM Fusion (BD biosciences)

Software	FACS Diva software (BD biosciences)
Cell population abundance	For in vitro culture experiments, more than 5,000,000 cells were isolated from the mouse lungs for each population through cell sorting. For the flow cytometry-based quantification of myofibroblasts, their progenitors, and smooth muscle cells in the lungs of postnatal mice, more than 20,000 cells were analyzed.
Gating strategy	Live cells were gated by FSC-A and SSC-A to exclude the debris. Dead cells were excluded by using SYTOX Blue dye. CD31(+), TER119(-), APC(-), Sytox blue(-) cells were isolated as endothelial cells, while CD31(-), TER119(-), APC(+), Sytox blue(-) cells were isolated as myofibroblasts and their progenitors.

☐ Tick this box to confirm that a figure exemplifying the gating strategy is provided in the Supplementary Information.
